# Immunohistochemical Expression of Autophagy-Related Proteins in Advanced Tubular Gastric Adenocarcinomas and Its Implications

**DOI:** 10.3390/cancers11030389

**Published:** 2019-03-19

**Authors:** Antonio Ieni, Roberta Cardia, Giuseppe Giuffrè, Luciana Rigoli, Rosario Alberto Caruso, Giovanni Tuccari

**Affiliations:** Department of Human Pathology in Adult and Developmental Age “Gaetano Barresi”, Section of Pathology, University of Messina, 98123 Messina, Italy; robertacardia87@gmail.com (R.C.); giuffre@unime.it (G.G.); lrigoli@unime.it (L.R.); rocaruso@unime.it (R.A.C.); tuccari@unime.it (G.T.)

**Keywords:** autophagy, immunohistochemistry, gastric cancer, tubular adenocarcinoma, grading

## Abstract

In neoplastic conditions, autophagy may act as a tumor suppressor avoiding the accumulation of damaged proteins and organelles or as a mechanism of cell survival promoting the tumor growth. Although ultrastructural analysis has been considered the traditional method to identify autophagy, some proteins such as microtubule-associated protein 1 light chain 3 (LC3A/B), Beclin-1 and activating molecule in Beclin-1-regulated autophagy protein-1 (AMBRA-1) may be considered as markers of autophagy-assisted cancerogenesis. Herein, we analyzed a cohort of advanced tubular gastric adenocarcinomas by the abovementioned immunohistochemical antisera; through immunohistochemistry, autophagy (A-IHC) is diagnosed when at least two out of the three proteins are positive in the samples. Immunostaining for LC3A/B, Beclin-1, and AMBRA-1 was exclusively found in neoplastic elements, but not in surrounding stromal cells. In detail, LC3A/B and Beclin 1 were expressed both in the cytoplasm and in the nucleus of the cancer cells, while AMBRA-1 was preferentially localized in the nucleus, mainly in high grade cases. LC3A/B, Beclin 1, and AMBRA-1 expression were positive in 18 (56.2%), 17 (53.1%), and 12 (37.5%) cases, respectively. The sensibility and specificity of LC3A/B and Beclin-1 ranged from 81.25% to 93.75%, with high efficiency (90.63%) for Beclin-1. Moreover, the ultrastructural autophagic index (AI) was also available in all cases. All high-grade cases documented a Ki-67 labelling index (LI) ≥ 30%, even if three low-grade cases revealed a high Ki-67 value; p53 positivity was encountered in 21/32 (65.62%) of cases, independently of the tumor grade. A statistically significant correlation among A-IHC and clinicopathological parameters such as grade, stage, clinical course, Ki-67 LI and AI was revealed. Univariate analysis documented a significant *p*-value for the same autophagic variables. Additionally, multivariate survival analysis identified the grade, AI and A-IHC as independent significant variables. Finally, the overall survival curves of all cases of gastric tubular adenocarcinoma were greatly dependent on A-IHC. Therefore, we suggest that autophagic-related proteins might be considered promising predictive prognostic factors of advanced gastric cancer. Further investigations may be required to determine whether new targeted therapies should be addressed to autophagy-related proteins.

## 1. Introduction

Autophagy is a fundamental dynamic catabolic process through which cells are able to recycle their own nutrients [1]. This process removes damaged organelles and cytoplasmic proteins and incapsulates them in a double-membrane vesicle called autophagosome [2,3,4,5]. Once fused with lysosome, this vesicle realizes a single membrane autolysosome [4,6,7]. Many researchers are still investigating the role of autophagy in cancer and in multidrug resistance [5,6,7,8,9]. Indeed, autophagy has been considered a process that frequently supports progression in experimental and human tumors [2,7,8].

Since the most traditional morphological approach to assess autophagy is electron microscopy (EM) [3,8,10], we have already performed an ultrastructural investigation to assess autophagy in a cohort of advanced gastric adenocarcinomas [7]. In particular, it has been demonstrated that autophagic vacuoles (AV) represent a frequent finding in high-grade tubular carcinomas compared with low-grade ones [7]. Consequently, autophagy has been strictly related to cellular differentiation as well as tumor progression [7]. 

Nevertheless, using a single marker to determine the autophagy status might prove unreliable. Autophagy should be assessed when neoplastic samples are positive for the immunoexpression of at least two autophagy-related proteins (A-IHC) [11,12]. Therefore, we applied three proteins that are closely associated and interact with each other, such as microtubule-associated protein 1 light chain 3 (LC3A/B), Beclin-1 and activating molecule in Beclin-1-regulated autophagy protein-1 (AMBRA-1). Furthermore, the abovementioned autophagy-related proteins may represent potential prognostic indicators as well as novel targets for cancer therapy, although it is well known that more than 30 autophagy-related (ATG) genes have been discovered [5,11]. Specifically, since LC3 has been considered a specific marker of autophagosome formation, it is widely monitored as an autophagy-related protein [12]. However, Beclin-1 is an essential modifier of the autophagic process and has been implicated in tumor development [12]. Finally, AMBRA-1 is a pro-autophagic protein that has been recently proven to take part in numerous regulatory mechanisms of the autophagy process [13].

In light of these suggestions, we thought it would be of interest to analyze a cohort of advanced tubular adenocarcinoma of the stomach with the abovementioned immunohistochemical antisera in order to verify any relationship among A-IHC, clinicopathological parameters and overall survival.

## 2. Results

The clinicopathological features as well as autophagic index (AI) and A-IHC of the low-grade and high-grade gastric tubular carcinoma cases are summarized in Table 1. Among 32 cases of surgically resected advanced tubular gastric carcinomas, according to the World Health Organization (WHO) classification, a two-tiered grading system distinguishing low-grade and high-grade carcinomas was applied: 15 cases were classified as low-grade, while 17 were classified as high-grade tubular adenocarcinomas. Histologically, the low-grade tubular adenocarcinoma group was characterized by well-formed glands, whereas the high-grade group included highly irregular glands and cells that were arranged singularly or in irregular clusters. 

The patient age ranged from 50 to 80 years (median 70 years), and the male to female (M–F) ratio was 20:12. All tumors were in advanced stage: 8 cases (stage II), 20 cases (stage III) and 4 cases (stage IV). 

The follow-up of patients ranged from 2 to 20 months (mean follow-up 11.1 months). During the follow-up observation period, the great majority (27 out 32) of the advanced tubular gastric adenocarcinomas died of the disease, while four low-grade and one high-grade adenocarcinoma patients were still alive at the end of the observation period. 

**Figure 1:**
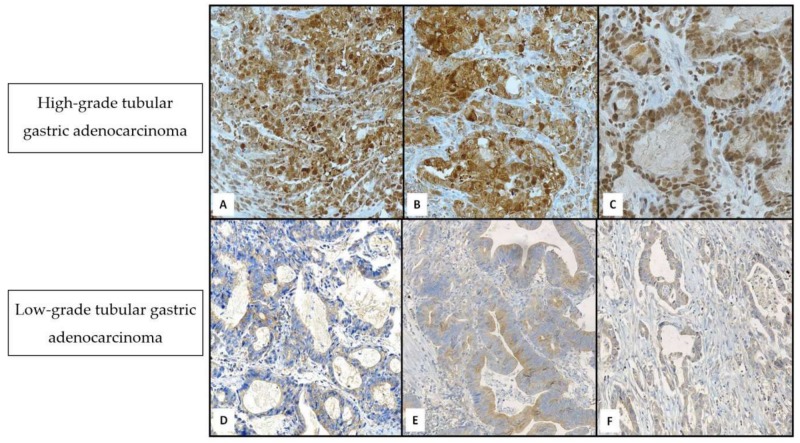
An evident strong and diffuse cytoplasmic and/or nuclear immunoreactivity was encountered in high-grade tubular gastric adenocarcinomas by LC3A/B (**A**, 160×), Beclin-1 (**B**, 160×) and AMBRA-1 (**C**, 200×); by contrast, a slight not uniform staining was found in low-grade cancer with LC3A/B (**D**, 120×), Beclin-1 (**E**, 120×) and AMBRA-1 (**F**, 140×). Nuclear Mayer’s haemalum counterstain.

Immunostaining for LC3A/B, Beclin-1 and AMBRA-1 were exclusively found in cancer cells, but not in surrounding stromal cells. LC3A/B (Figure 1A) and Beclin 1 (Figure 1B) were expressed both in the cytoplasm and in the nucleus of neoplastic elements, mainly in high grade cancers; AMBRA-1 was preferentially localized in the nucleus (Figure 1C). After having selected tumors by the proposed immunoreactive score (0–3 = negative; 4–6 = positive), LC3A/B, Beclin 1, and AMBRA-1 expression were positive in 18 (56.2%), 17 (53.1%), and 12 (37.5%) cases, respectively. Interestingly, in the low-grade tubular carcinomas only two cases exhibited an immunopositive cytoplasmic reaction for LC3A/B (Figure 1D) and Beclin 1 (Figure 1E), whereas four different low-grade cases showed AMBRA-1 positive expression (Figure 1F). In contrast, in the high-grade group 15 cases documented LC3A/B and Beclin 1 immunoreactivity, while in eight cases additional AMBRA-1 immunopositivity was evident. Therefore, on the basis of Masuda’s criteria, A-IHC was determined [12]. As a result, 17/32 (53.1%) advanced tubular gastric cancers exhibited immunohistochemical evidence of at least two autophagy-related proteins (LC3 A/B and Beclin 1). A high significant correlation between the A-IHC and grade, stage, clinical course as well as AI was revealed (Table 1). 

In terms of the growth fraction, all high-grade cases showed a Ki-67 LI ≥30%, even if three low-grade cases also revealed a high Ki-67 value. The Ki-76 LI exhibited a strong significant relationship with A-IHC. Finally, p53 positivity was encountered, either focal or diffuse, in 21/32 (65.62%) of cases, but any association with A-IHC, AI and other parameters was appreciable. 

The sensibility, specificity and efficiency (expressed as a percentage of what could ideally be expected, with 100% representing the ideal case) of each immunohistochemical expression of autophagy-related proteins were evaluated. LC3A/B and Beclin-1 exhibited the greatest sensibility (both 93.75%), while Beclin-1 was characterized by the highest specificity (87.50%) and efficiency (90.63%). Table 2 shows the analytical data concerning each antiserum.

The univariate analysis relative to cancer-specific mortality using the Mantel–Cox log-rank test in gastric carcinoma GC is summarized in Table 3. In detail, the grade, stage, Ki67 LI, AI and A-IHC appeared to be prognostic significant parameters with a high *p*-value. Moreover, using Cox multivariate analysis, grade, AI and A-IHC emerged as independent prognostic variables for the GC patients (Table 4).

**Figure 2:**
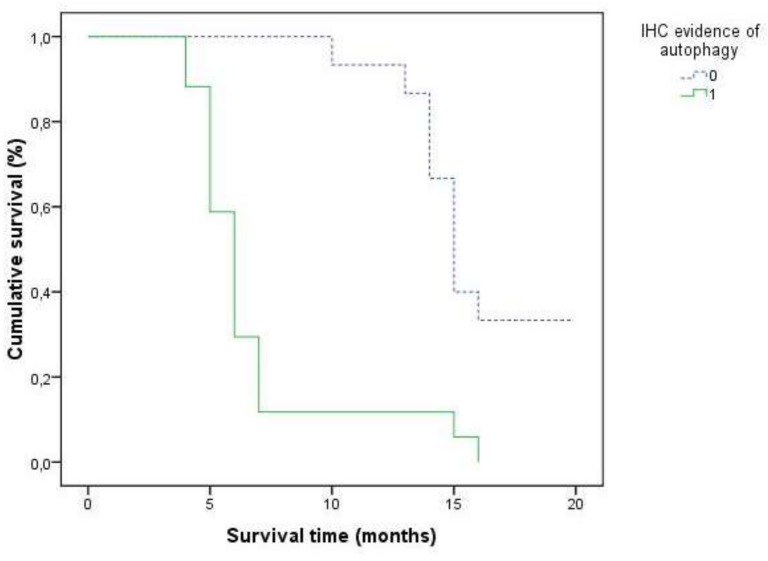
Overall survival curves of all cases of gastric tubular adenocarcinomas in relation to immunohistochemical evidence of autophagy.

Finally, the survival curves of all patients, as illustrated in Figure 2, showed that a different overall survival rate was evident between positive and negative cases, depending on the autophagy-related proteins. 

## 3. Discussion

Generally speaking, autophagy has been considered an adaption mechanism utilized by tumor cells to survive in a hostile stromal microenvironment [14]; in fact, experimental studies have shown enhanced autophagy in hypoxic neoplastic areas [15]. Nevertheless, mechanisms of autophagy in the tumorigenesis, progression and prognosis in gastric carcinomas are still unclear. 

Herein we investigated the expression of some autophagy-related proteins in advanced tubular gastric cancer alongside a comparative analysis of ultrastructural findings for authophagic vacuoles and clinicopathological parameters. For this purpose, Masuda’s criteria was taken into consideration [12], and the autophagy status was determined utilizing LC3A/B, Beclin-1 and AMBRA-1 in order to show their potential overexpression in relation to the formation of autophagosomes, as well as their significance in terms of tumor progression and patient outcome. 

It is well known that LC3 A/B represents one of the most reliable and widely used biomarkers for autophagy, since its expression is a prognostic factor in various human cancers, including gastric adenocarcinoma [16,17], colorectal cancers [18,19,20,21], melanoma [22], astrocytoma [23], esophageal cancer [24], oral squamous cell carcinoma [25] and hepatocellular carcinoma [26]. As Beclin-1 interacts with members of the anti-apoptotic Bcl-2 protein family [27], it has been suggested that loss of Beclin-1 expression defines poor prognosis, presumably by enhancing anti-apoptotic pathways [28]. The overexpression of Beclin-1 also defines subgroups of tumors with aggressive clinical behavior [29], presumably by promoting autophagy [28]. Additionally, Beclin-1 acts as a scaffold for the structure of the phosphatidylinositol 3 kinase (PI3K) complex and its levels are dysregulated in advanced stage tumors, as revealed in breast cancer, lung cancer and lymphoma, whereas conflicting data between favorable and poor prognostic values have been shown in colorectal cancer [28,30]. Finally, AMBRA-1 is implied in autophagy as well as cell growth, cell death, embryonic development and carcinogenesis [13]; moreover, in pancreatic ductal adenocarcinoma, cholangiocarcinoma and prostate carcinoma, its poor prognostic value has been inferred [28,31]. However, high levels of AMBRA-1 protein have been found in GC patients and they appeared significantly related to depth of invasion and lymph node metastasis [32].

In the present study, we utilized the three abovementioned autophagy-related proteins, also defining a quantitative score (0–3 = negative; 4–6 = positive) to select the degree of autophagy in our casuistry of advanced tubular gastric carcinomas. Interestingly, the high-grade group of advanced adenocarcinomas revealed a significant immunopositivity in comparison to the low-grade group. In particular, LC3 A/B, Beclin-1 and AMBRA-1 expression was positive in 56.2%, 53.1% and 37.5% of cases, respectively. However, the immunopositive rate for LC3 A/B as well as Beclin 1 was not surprising, since they have been highly expressed in various types of cancer cells with autophagy-positive status [30]. On the other hand, the immunoexpression of AMBRA-1 antiserum found in our study was lower than that reported by Qu et al. (37.5% vs. 55.2%) [32]. In this regard, the explanations with the highest level of consensus may be related to different ethnical geographical populations, the selection of neoplastic gastric histotypes, validation at the ultrastructural level of reported casuistries, and alternative uses of tissue microarray construction in whole section testing in immunohistochemical procedures.

Assuming that the “autophagy signature” (A-IHC) was documented when the GC showed positive immunoreactivity for at least two antibodies, the immunoreactive rate observed was 53.1%, which reflects a high significant correlation with the grade, stage, growth fraction, clinical course as well as AI. Moreover, in multivariate analysis, A-IHC emerged as an independent prognostic variable together with the grade as well as AI. Consequently, it is not surprising that different rates of overall survival were revealed between positive and negative cases for the expression of autophagy-related proteins. 

The suggested hypothesis that autophagy may be associated with aggressive clinical behavior, such as vessel invasion, lymph node disease, and hepatic metastasis in gastric cancer, has been already proposed in pancreatic cancer [31] and oral cell carcinoma [30]. However, our data strongly supports the idea that “autophagy signature” represents a marker that promotes the progression of gastric cancer, and it has been previously reported as an independent prognostic factor in poor survival patients with advanced gastric cancer [8]. In addition, some studies have assessed prognostic value in GC and pancreatic ductal carcinomas utilizing 5–10 autophagy markers, demonstrating a correlation with the final outcome [16,31]. Among these latter markers, the best prognostic performance was attributed to Beclin-1 [16,31]; however, in our study Beclin-1 exhibited the highest sensibility, specificity and efficiency. Finally, among other autophagic-related proteins, p62 (multi-functional signaling molecule for cell survival and cell death) has been localized at the membranes of autophagosomes, and it has been independently associated with worse prognosis and poor survival in patients with gastric cancer [32].

## 4. Materials and Methods

The study was conducted in accordance with Good Clinical Practice guidelines and the Declaration of Helsinki (1975, revised in 2013); its retrospective nature did not require any informed consent, even if written informed consent had been obtained from each patient before surgical procedures. The clinical information had been retrieved from the patients’ medical records and pathology reports. Patients’ initials or other personal identifiers did not appear in any image. Finally, all samples were anonymized before histology and immunohistochemistry. Therefore, no further ethical approval was necessary to perform the study.

### 4.1. Case Selection

From the archives of the Department of Human Pathology of Adult and Evolutive Age (University of Messina, Messina, Italy), a cohort of 32 cases of surgically resected gastric tubular type carcinomas were selected; twenty-five of them had been previously analyzed by EM and the morphological results have been already reported elsewhere [7]. Successively, seven additional cases were routinely processed for both light and electron microscopic observations.

Clinical and pathological parameters such as age, gender, tumor site, pTNM stage, grade and clinical course regarding all cases of tubular-type gastric carcinomas (WHO Classification of Tumors of the Digestive System, Fourth Edition, 2010) were collected. 

An autophagic index according to Ma et al. [33] was achieved for all cases, whereby high-powered micrographs of 20–35 single neoplastic cells from random distinct ten fields in each section were obtained. The mean number of autophagic vacuoles per cell were scored for each tumor, in accordance to previous reports [7]. The mean AV/cell number was recorded for each case; a value of 4 AV/cell was considered the cut-off to distinguish the low and high autophagic indexes. 

### 4.2. Immunohistochemistry

For immunohistochemical procedures, 5-micron thick sections obtained from corresponding tissue-blocks were deparaffinized, then washed in descending alcohol scale, treated by 3% hydrogen peroxide for 10 min, washed again in deionized water three times and incubated with normal sheep serum to prevent unspecific adherence of serum proteins for 30 min at room temperature. Subsequently, sections were washed with deionized water and incubated for 30 min at 37 °C with commercially obtained primary polyclonal rabbit anti-human antisera against Beclin-1 (working dilution 1:250; Abcam, Cambridge, MA, USA), AMBRA1 (working dilution 1:250; Abcam, Cambridge, MA, USA) and LC3A/B (working dilution 1:100; Abcam, Cambridge, MA, USA). Next, the sections were washed three times with PBS and incubated with a biotinylated goat anti-rabbit IgG secondary antibody (1:300; Abcam) for 20 min at room temperature, subsequently incubated with horseradish peroxidase-labeled secondary antibody for 30 min and developed with diaminobenzidine tetrahydrochloride and counterstained with hematoxylin using the ULTRA Staining system (Ventana Medical Systems, Tucson, AZ, USA). Negative controls were obtained omitting the specific antisera and substituting PBS for the primary antibody.

The immunoreactivity of Beclin1, AMBRA1 and LC3A/B was evaluated according to the intensity and percentage of positively stained cells, as elsewhere reported [34]. The cytoplasmic immunostaining intensity was rated as follows: 0, negative; 1, weak; and 2, strong. The percentage of positively stained cells was graded as follows: grade 0, 0–5%; grade 1, >5–25%; grade 2, >25–50%; grade 3, >50–75%; and grade 4, >75–100% for all antibodies. The immunohistochemical staining samples were independently scored by two pathologists (AI and GT), who were blinded to patient outcomes and other clinical findings, using a Zeiss Axioskop microscope (Carl Zeiss Microscopy GmbH, Jena, Germany) at 40× objective magnification. The interobserver agreement for immunohistochemistry staining had a kappa value of ranging from 0.73–0.80 (substantial agreement) for the antisera.

The immunoreactive score was calculated by adding the staining intensity score and the percentage score of positively stained cells (0–6). Tumors with an immunoreactive score of 0–3 were classified as negative, and those with a score of 4–6 were classified as positive. Autophagy was defined when samples were positive for at least two out the three protein expressions [12]. The sensibility, specificity and efficiency (expressed as a percentage of what could ideally be expected, with 100% representing the ideal case) of each immunohistochemical expression of autophagy-related proteins were evaluated.

In order to determine the growth fraction of each neoplastic sample, parallel sections were obtained from the same tissue blocks, and Ki-67 antigen was unmasked by antigen retrieval pre-treatment performed with three changes in 0.01 M citrate buffer (pH 6.0) in a microwave oven at 750 W. Ki-67 antiserum (clone MIB-1, dilution 1:100, Dako Corp., Glostrup, Denmark) was applied for 30 min at room temperature. The Ki-67 labeling index (LI) was calculated as the mean percentage by counting the stained nuclei of 1000 tumor cells in three representative neoplastic fields. All degrees of nuclear staining intensity were taken into consideration. The median Ki-67 LI value (30%) was utilized as the cut-off point to define low and high Ki-67 expression.

In addition, serial sections were immunostained by p53 (clone DO-7, dilution 1:250, Novocastra Corp., Newcastle Upon Tyne, United Kingdom). The p53 mutation status was defined as positive in the presence of nuclear staining, while cytoplasmic staining was considered negative. Gastric carcinomas were considered focally positive when staining was present in 10–50% on tumor cells and diffusely positive when >50% of neoplastic elements were immunoreactive.

### 4.3. Statistical Analysis

Statistical evaluation was performed using the SPSS version 13.0 software package (SPSS, Inc., Chicago, IL, USA). The association between LC3A/B, Beclin-1 and AMBRA-1 expression and clinicopathological features (age, gender, tumour site, pTNM stage, grade, Ki-67 LI and p53) was analyzed using the Chi-square (χ^2^) or Fisher exact test. Cancer-specific survival analysis was performed by the Kaplan–Meier method, and for comparison of the survival curves, the Mantel–Cox log-rank test was used. A multivariate analysis (Cox regression model) was utilized to determine the independent effects of variables on overall survival. A value less than 0.05 was considered statistically significant.

## 5. Conclusions

In our opinion, the expression of an immunohistochemical autophagic signature may be considered a potential prognostic tool in tubular adenocarcinomas of the stomach. Indeed, A-IHC, together with other clinicopathological characteristics, such as grade, stage and Ki-67 LI, identifies a more accurate rationale for the function of autophagy in tumor progression. Additionally, the role of A-IHC in advanced GC is greatly supported by multivariate analysis, in which this parameter emerged as the independent variable, similar to AI and tumor grade. Therefore, in terms of final outcome, patients affected by GC with an immunopositive autophagic signature exhibited the worst prognosis. Nevertheless, some biases, such as the small patient cohorts, the absence of standard techniques and the limited number of autophagy-related markers available, may represent a possible explanation for the conflicting reports about the prognostic role of autophagy protein expression. 

Finally, several therapeutic agents modulating autophagy have been developed in colorectal cancer, showing promising results either alone or in association with other drugs [35]. Unfortunately, in advanced gastric adenocarcinomas many patients died during follow-up with a generally worse outcome. Consequently, additional investigations are required to better explain the relationships between this neoplastic entity and autophagy, and to recognize potentially new beneficial chemotherapeutic targeted agents 

## Figures and Tables

**Table 1:** Clinico-pathological parameters and corresponding immunoexpression autophagy-related proteins in gastric carcinomas cases.

Parameter	No.	LC3AB (%)	*p* Value	Beclin-1 (%)	*p* Value	AMBRA-1(%)	*p* Value	A-IHC (%)	*p* Value
Sex			NS		NS		NS		NS
M	20	12 (60.0)		12 (60.0)		9 (45.0)		12 (60.0)	
F	12	6 (50.0)		5 (41.7)		3 (25.0)		5 (41.7)	
Location			NS		NS		NS		NS
PT	4	1 (25.0)		1 (25.0)		1 (25.0)		1 (25.0)	
MT	11	8 (72.7)		7 (63.6)		5 (45.5)		7 (63.6)	
DT	14	6 (42.9)		6 (42.9)		5 (35.7)		6 (42.9)	
ES	3	3 (100)		3 (100)		1 (33.3)		3 (100)	
Grade			<0.001		<0.001		NS		<0.001
Low	15	2 (13.3)		2 (13.3)		4 (26.7)		2 (13.3)	
High	17	16 (94.1)		15 (88.2)		8 (47.1)		15 (88.2)	
Stage			0.041		0.047		NS		0.047
II	8	2 (25,0)		2 (25,0)		1 (12.5)		2 (25,0)	
III	20	12 (60.0)		11 (55.0)		8 (40.0)		11 (55.0)	
IV	4	4 (100)		4 (100)		3 (75.0)		4 (100)	
Clinical course			0.01		0.015		NS		0.015
Alive	5	0 (0)		0 (0)		0 (0)		0 (0)	
Dead	27	18 (66.7)		17 (63.0)		12 (44.4)		17 (63.0)	
Ki67			<0.001		<0.001		NS		<0.001
<30%	13	2 (15.4)		1 (7.7)		4 (30.8)		1 (7.7)	
≥30%	19	16 (84.2)		16 (84.2)		8 (42.1)		16 (84.2)	
p53			NS		NS		NS		NS
Negative	11	7 (63.6)		6 (54.5)		3 (27.3)		6 (54.5)	
Positive	21	11 (52.4)		11 (52.4)		9 (42.9)		11 (52.4)	
AI			<0.001		<0.001		NS		<0.001
<4 AV/cell	16	3 (18.8)		2 (12.5)		5 (31.3)		2 (12.5)	
≥4 AV/cell	16	15 (93.8)		15 (93.8)		7 (43.8)		15 (93.8)	

NS: not significant; A-IHC: immunohistochemical evidence of autophagy, M = male; F = female; PT = proximal third; MT = middle third; DT = distal third; ES = entire stomach; AI = autophagic index.

**Table 2:** Performance of autophagy-related proteins by immunohistochemistry in advanced tubular gastric adenocarcinomas.

A-IHC Analysis	LC3A/B	Beclin-1	AMBRA-1
Sensibility	93.75%	93.75%	43.75%
Specificity	81.25%	87.50%	68.75%
Efficiency	87.50%	90.63%	56.25%

**Table 3:** Prognostic parameters examined in gastric tubular adenocarcinomas: A univariate analysis of cancer-specific mortality using the Mantel-Cox log-rank test.

Parameter	X^2^	df	*p* Value
Sex	0.075	1	NS
Grade	19.321	1	0.000
Stage	6.253	1	0.012
Ki67 status	8.278	1	0.004
p53 status	0.470	1	NS
AI	10.842	1	0.001
A-IHC	18.883	1	0.000

NS: not significant; df: degrees of freedom; AI: autophagic index; A-IHC: immunohistochemical evidence of autophagy.

**Table 4:** Multivariate survival analysis using the Cox regression model in gastric tubular adenocarcinomas.

Parameter	β	SE	Exp(β)	*p* Value
Grade	2.361	1.084	10.601	0.029
AI	1.327	0.643	3.770	0.039
A-IHC	2.922	0.885	18.573	0.001

AI: autophagic index; A-IHC: immunohistochemical evidence of autophagy; β: regression coefficient; SE: standard error; Exp(β): ratio of risk.
